# Factors Associated with Prolonged Prehospital On-Site Time in Adult Trauma Patients: A Nationwide Observational Study in Japan

**DOI:** 10.31662/jmaj.2025-0290

**Published:** 2026-02-06

**Authors:** Shunichi Otaka, Takuyo Chiba, Takamichi Nakajima, Takashi Shiga

**Affiliations:** 1 International University of Health and Welfare Narita Hospital, Chiba, Japan

**Keywords:** trauma, prehospital, database

## Abstract

**Introduction:**

Time elapsed since trauma onset is a critical determinant of prognosis in trauma patients. However, factors behind prolonged prehospital on-site time remain unclear. This study aimed to identify associated factors in trauma cases.

**Methods:**

Using a nationwide Japanese database, adults who experienced trauma and were treated between January 2004 and May 2019 were identified. Multivariable logistic regression with multiple imputations for missing data was performed to compare characteristics of patients with shorter and longer prehospital on-site times.

**Results:**

Data from 150,215 patients were included. Multivariable logistic regression with multiple imputations for missing data revealed that longer prehospital on-site time was associated with younger age, male sex (odds ratio [OR] 1.14; 95% confidence interval [CI] 1.11-1.16), weekends and holidays (OR, 1.06; 95% CI, 1.03-1.08), alcohol consumption (OR, 1.31; 95% CI, 1.26-1.36), fall (OR, 1.07; 95% CI, 1.05-1.10), other blunt trauma (OR, 1.12; 95% CI, 1.07-1.18), suicide (OR, 1.20; 95% CI, 1.13-1.27), violence (OR, 1.33; 95% CI, 1.20-1.46), hypotension (OR, 1.15; 95% CI, 1.09-1.22), higher Revised Trauma Score (OR, 1.21; 95% CI, 1.20-1.23), Abbreviated Injury Scale (AIS) category 6 (spine) ≥ 3 (OR, 1.21; 95% CI, 1.17-1.25), AIS category 7 (upper extremities) ≥ 3 (OR, 1.15; 95% CI, 1.10-1.21), immobilization (OR, 1.25; 95% CI, 1.22-1.28), intravenous line placement (OR, 1.27; 95% CI, 1.17-1.37), intubation (OR, 1.44; 95% CI, 1.26-1.64), mental disease (OR, 1.07; 95% CI, 1.02-1.13), chronic kidney disease undergoing hemodialysis (OR, 1.12; 95% CI, 1.03-1.22), and malignancy (OR, 1.11; 95% CI, 1.04-1.18).

**Conclusions:**

Several factors associated with prolonged prehospital on-site time were identified. Interventions targeting these factors may help shorten prehospital on-site time.

## Introduction

Timely definitive treatment is critical to the prognosis of individuals who experience trauma, and delays during the prehospital period―particularly before arrival at the hospital―have been shown to adversely affect outcomes in this patient population ^(1), (2), (3), (4), (5), (6)^. Prehospital time encompasses several key intervals, including the interval between the emergency call and arrival at the scene, time devoted to treatment at the scene, hospital selection, and transportation to the hospital. Though advances in transportation infrastructure and the use of helicopters have reduced some of these intervals, the time spent at the scene remains highly variable, depending on factors including injury severity and management, and challenges in selecting the appropriate hospital ^(7), (8)^.

For severely injured patients, balancing prehospital on-site treatment with prompt transport is critical because unnecessary delays at the scene can negatively affect prognosis. Conversely, prolonged prehospital on-site time for patients with only minor injuries can delay responses to subsequent or other more serious emergencies, posing broader public health concerns ^(9)^. A previous study demonstrated that factors such as male sex, injury severity, nighttime incidents, and holidays contribute to difficulties in hospital selection for traffic-related trauma cases ^(10)^. However, the study did not specifically analyze the duration of prehospital on-site time. Furthermore, it did not adequately adjust for confounding factors such as alcohol use, specific type or cause of trauma (e.g., traffic accident, penetrating injury, accidental injury, self-harm, and violence), detailed trauma severity, on-site treatment details, or medical history. For example, patients with a history of psychiatric disorders, malignancies, or those undergoing dialysis treatment may face resistance from medical institutions that are unable to manage treatments for pre-existing conditions other than trauma, which could lead to difficulties in smooth acceptance. Given the potential for stigma associated with alcohol use and mental illness among medical staff, we hypothesized that these factors could influence emergency medical team treatment decisions and the acceptance of patients by medical institutions, thereby leading to prolonged on-site time ^(11), (12), (13)^. In fact, a previous study suggested a possible association between psychiatric disorders and prolonged on-site time exceeding 30 minutes ^(14)^. However, that analysis grouped psychiatric disorders with other conditions such as eye and skin diseases, obscuring the specific relationship between psychiatric disorders alone and on-site time.

As such, the present study aimed to identify factors associated with prolonged prehospital on-site time during ambulance transport in trauma patients using information obtained from a nationwide database in Japan. By addressing gaps in previous research and focusing on prehospital delays, particularly through the inclusion of factors such as alcohol use, detailed trauma severity, on-site treatment details, and medical history, including mental health conditions, this study aimed to identify factors associated with prehospital delays that could potentially be addressed through clinical intervention or system improvement.

## Materials and Methods

### Emergency medical service system in Japan

In 2019, Japan had 726 fire stations equipped with dispatch centers. The Emergency Medical Service (EMS) is managed by the Fire and Disaster Management Agency of Japan. All patients were transported to the hospital, except in cases involving decapitation, incineration, decomposition, or rigor mortis. Within a prehospital environment, the EMS team can perform the following procedures for patients with trauma: capture and evaluate an initial electrocardiogram; administer oxygen; immobilize the patient using a cervical collar or backboard; perform chest compressions; and use an automated external defibrillator. Under remote medical direction, EMS can perform the following procedures for trauma patients who are in shock or experiencing cardiopulmonary arrest: placing a peripheral intravenous line; administering Ringer’s lactate solution; providing intravenous adrenaline (epinephrine); and establishing an advanced airway using an endotracheal tube, laryngeal mask airway, esophageal gastric tube airway, or combi tube.

### Data collection

Patient data for this retrospective cohort study were collected from a nationwide trauma database in Japan, otherwise known as the Japan Trauma Data Bank (JTDB), which has been described in detail elsewhere ^(15)^. Briefly, the registry is managed by the Japanese Association for the Surgery of Trauma (Trauma Surgery Committee) and the Japanese Association for Acute Medicine (Committee for Clinical Care Evaluation). More than 80% of tertiary care hospitals in Japan participate in the database. Data collection began in January 2004 and is ongoing. The database includes individual information, chronological data regarding the event, comorbidities, prehospital vital signs, information regarding the trauma (type, cause, and severity), and interventions before hospital arrival.

The following variables were collected: age (years); sex; year; month; day of the week; time of day; call time zone (daytime and nighttime); alcohol consumption; type of trauma (traffic accident [i.e., motor vehicle collision], fall, other blunt trauma [blunt injuries that are not classified as traffic-related or falls. Examples include injuries caused by sports activities, being struck by falling objects, and similar mechanisms], penetrating trauma); cause of trauma (accident, occupational injury, suicide, violence, unclassified/rare mechanisms, unknown); presence of hypotension (systolic blood pressure < 90 mmHg); severity of trauma (revised trauma score [RTS] ^(16)^, abbreviated injury scale [AIS] ^(17)^); interventions before arrival to the hospital (oxygen administration, immobilization with cervical collar or backboard, chest compression, intravenous line placement, defibrillation, and intubation); comorbidities (mental disease, chronic kidney disease undergoing hemodialysis, malignancies [specifically, malignancy requiring ongoing treatment at the time of the event], diabetes mellitus, ischemic heart disease, cerebrovascular disease, dementia/intellectual disability); and prehospital on-site time (time spent on-site between arrival to and departure from the scene).

Age was subdivided into five categories: 19-59, 60-69, 70-79, 80-89, and > 89 years. The year of the case was divided into five categories: 2004-2007, 2008-2010, 2011-2013, 2014-2016, and 2017-2019. Months were categorized into four groups: January to March; April to June; July to September; and October to December. Time was divided into daytime (09:00-16:59) and nighttime (17:00-08:59) ^(10)^. The AIS was divided into nine anatomical regions, as follows: AIS1 (head); AIS2 (face); AIS3 (neck); AIS4 (thorax); AIS5 (abdomen/pelvic content); AIS6 (spine); AIS7 (upper extremities); AIS8 (lower extremities); and AIS9 (external, burns/other trauma). An AIS score ≥ 3 was considered to be severe. The AIS coding version ‘AIS 90 Update 98’ was used in the database employed in this study ^(18)^. Prehospital interventions were identified from the dataset; however, the timing of the intervention―whether it was performed at the trauma scene or during transport―was not available. Analyses were adjusted for injury severity as well as other relevant patient characteristics to reduce potential confounding and better reflect patient heterogeneity.

We selected variables for the multivariable model based on their clinical relevance and previous literature, focusing on factors that are considered to potentially influence prehospital time ^(10), (19), (20)^. To avoid introducing selection bias, we carefully included only those variables that could act as potential confounders or independent predictors, while avoiding variables that might serve as mediators in the causal pathway. Furthermore, we examined the correlations among variables and calculated variance inflation factors (VIF) to ensure that there was no significant multicollinearity among variables included in the model (Supplementary Table 1).

### Patient selection and study outcome

Data regarding adults (age >18 years) who experienced trauma and who arrived at the hospital by ambulance between January 1, 2004, and May 28, 2019, were collected; cases involving physician-staffed ambulances, including doctor cars, were excluded. “Prehospital on-site time” was defined as the interval between EMS arrival at and departure from the scene. Patients with prehospital on-site times > 120 minutes were considered to have unrealistic prehospital on-site time records. Patients with unrealistic on-site prehospital time data and those with missing prehospital time data were excluded.

The median prehospital on-site time was calculated to divide the patients into two groups: shorter prehospital on-site time (shorter on-site time) and longer prehospital on-site time (longer on-site time). Variables between the groups were compared, and the factors that contributed to prolonged prehospital on-site time were investigated.

### Statistical analysis

Characteristics of the study population were analyzed first. Continuous variables are expressed as median and interquartile range (IQR), whereas categorical variables are expressed as frequency and percentage. Second, univariable logistic regression was performed to compare variables between the shorter on-site time versus the longer on-site time groups. Multivariable logistic regression with multiple imputations for missing data was performed to compare variables between the shorter on-site time versus longer on-site time groups to account for bias caused by missing data. In this study, missing values were addressed by generating 20 complete datasets using multiple imputations with the chained-equation method and 20 iterations, assuming that the data were missing at random ^(21)^. All of the variables used in the multivariate analysis were included in the imputation process. The odds ratio (OR) and corresponding 95% confidence interval (CI) were calculated according to Rubin’s rule. To confirm the robustness of the results, we conducted additional analyses stratified by time period (2004-2019) and sensitivity analyses excluding patients with cardiac arrest at the scene, with a different cutoff value for on-site time (30 minutes), and by performing a complete-case analysis excluding all patients with missing data ^(22)^.

Results are reported as OR with corresponding 95% CI and two-sided *p*-value. All statistical tests were two-sided and differences with p < 0.05 were considered to be significant. Statistical analyses were performed using STATA Release 16.0 (StataCorp LLC, College Station, TX, USA).

### Ethics

This study was approved by the Institutional Review Board of the International University of Health and Welfare, Narita Hospital (Chiba, Japan; Approval number: 22-Im-008; June 28, 2022). Given the retrospective design of the study and the use of anonymized patient data, requirements for informed consent were waived.

## Results

A total of 203,166 adults who experienced trauma were registered in the JTDB; after applying the exclusion criteria, data from 150,215 were included in the present study. The baseline characteristics of the study population are summarized in Table 1. Several variables had missing data. For example, there was a 33.1% missing data rate for alcohol consumption and a 15.1% missing data rate for RTS. The median patient age was 64 years, and there were more males than females.

**Table 1.** Characteristics of the Study Population.

Variables	Total (n = 150,215)
Age: years, median (IQR)	64 (44-78)
Age groups: years, n (%)	
19-59	65,020 (43.2)
60-69	24,165 (16.1)
70-79	27,303 (18.2)
80-89	26,038 (17.3)
89 <	7,689 (5.1)
Sex, n (%)	
Male	92,263 (61.4)
Female	57,921 (38.6)
Missing	31 (<0.1)
Year, n (%)	
2004-2007	9,254 (6.2)
2008-2010	16,791 (11.2)
2011-2013	34,157 (22.7)
2014-2016	49,239 (32.8)
2017-2019	40,161 (26.7)
Missing	613 (0.4)
Month, n (%)	
January-March	36,636 (24.4)
April-June	35,359 (23.5)
July-September	36,954 (24.6)
October-December	40,618 (27.0)
Missing	648 (0.4)
Days of the week, n (%)	
Weekdays	99,840 (66.5)
Weekends/Holidays	50,353 (33.5)
Missing	22 (<0.1)
Time, n (%)	
Daytime	61,315 (40.8)
Nighttime	87,651 (58.4)
Missing	1,249 (0.8)
Alcohol consumption, n (%)	
Yes	16,267 (10.8)
No	84,244 (56.1)
Missing	49,704 (33.1)
Type of trauma, n (%)	
Traffic accident	59,862 (39.8)
Fall	72,659 (48.4)
Other blunt trauma	10,086 (6.7)
Penetrating trauma	5,039 (3.4)
Missing	2,569 (1.7)
Cause of trauma, n (%)	
Accident	124,235 (82.7)
Occupational injury	8,441 (5.6)
Suicide	10,307 (6.9)
Violence	2,076 (1.4)
Unclassified/rare mechanisms	1,334 (0.9)
Unknown/missing	3,822 (2.5)
**Prehospital vital signs**	
Systolic blood pressure: mmHg, median (IQR)	139 (118-160)
Hypotension (< 90 mmHg)	7,378 (4.9)
Missing, n (%)	16,400 (10.9)
**Severity of trauma**	
Revised trauma score, median (IQR)	7.84 (7.26-7.84)
Missing, n (%)	22,721 (15.1)
AIS	
AIS1 > 3, n (%)	47,109 (31.4)
AIS2 > 3, n (%)	1,219 (0.8)
AIS3 > 3, n (%)	847 (0.6)
AIS4 > 3, n (%)	34,340 (22.9)
AIS5 > 3, n (%)	7,046 (4.7)
AIS6 > 3, n (%)	15,316 (10.2)
AIS7 > 3, n (%)	7,551 (5.0)
AIS8 > 3, n (%)	41,906 (27.9)
AIS9 > 3, n (%)	78 (0.1)
**Interventions before hospital arrival**	
Oxygen	69,902 (46.5)
Immobilization	82,518 (54.9)
Chest compression	5,982 (4.0)
Intravenous line placement	3,066 (2.0)
Defibrillation	312 (0.2)
Intubation	1,134 (0.8)
Missing	9,858 (6.6)
**Comorbidities**	
Mental disease	10,028 (6.7)
Chronic kidney disease on hemodialysis	2,191 (1.5)
Malignancy	4,079 (2.7)
Diabetes mellitus	16,541 (11.0)
Ischemic heart disease	5,789 (3.9)
Chronic heart disease	2,985 (2.0)
Cerebrovascular disease	7,786 (5.2)
Dementia/mental retardation	7,929 (5.3)
On-site time, median (IQR)	14 (10-20)

AIS: abbreviated injury scale; IQR: interquartile range.

**Figure 1.**
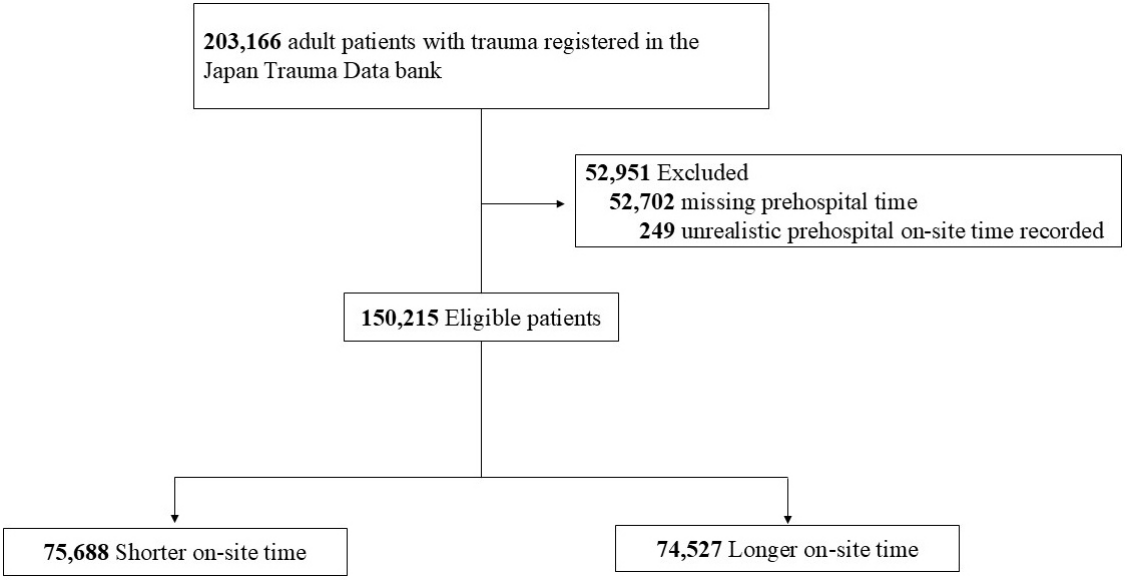
Flowchart of patient selection.

A flow diagram illustrating the patient selection process is presented in Figure 1. The median on-site prehospital time was 14 minutes; accordingly, 75,688 patients were assigned to the shorter on-site time group (prehospital on-site time <15 minutes) and 74,527 to the longer on-site time group (prehospital on-site time >14 minutes).

Results of univariable logistic regression analysis comparing factors between the shorter on-site time versus longer on-site time groups are summarized in Table 2. According to analysis, the following factors were associated with longer on-site time: younger age; male sex; more recent trauma; weekends and holidays; alcohol consumption; fall; other blunt trauma; penetrating trauma; violence; higher RTS score, AIS6 ≥ 3, AIS7 ≥ 3, immobilization, mental disease; chronic kidney disease undergoing hemodialysis; malignancy; and diabetes mellitus. Injury during the daytime was associated with shorter on-site time.

**Table 2.** Univariable Logistic Regression Analysis of Variables Associated with the Length of On-Site Time.

Variables	Odds Ratio	95% confidence interval			p-Value
Age groups: years					
19-59			Reference		
60-69	0.98	0.95	to	1.01	0.23
70-79	0.92	0.90	to	1.91	<0.001
80-89	0.88	0.85	to	0.90	<0.001
89 <	0.80	0.76	to	0.83	< 0.001
Sex					
Male	1.18	1.16	to	1.21	< 0.001
Year					
2004-2007			Reference		
2008-2010	1.17	1.11	to	1.23	< 0.001
2011-2013	1.26	1.21	to	1.32	< 0.001
2014-2016	1.49	1.43	to	1.56	< 0.001
2017-2019	1.53	1.46	to	1.60	< 0.001
Month					
January-March			Reference		
April-June	0.89	0.86	to	0.91	< 0.001
July-September	0.85	0.83	to	0.99	< 0.001
October-December	0.91	0.88	to	0.93	< 0.001
Days of the week					
Weekdays			Reference		
Weekends/Holidays	1.08	1.06	to	1.10	< 0.001
Time					
Daytime	0.75	0.74	to	0.77	< 0.001
Alcohol consumption					
Yes	1.44	1.39	to	1.49	< 0.001
Type of trauma					
Traffic accident			Reference		
Fall	1.06	1.03	to	1.08	< 0.001
Other blunt trauma	1.09	1.04	to	1.13	< 0.001
Penetrating trauma	1.16	1.09	to	1.22	< 0.001
Cause of trauma					
Accident			Reference		
Occupational injury	0.96	0.92	to	1.00	0.08
Suicide	0.91	0.87	to	0.95	< 0.001
Violence	1.45	1.33	to	1.58	< 0.001
Unclassified/rare mechanisms	1.21	1.08	to	1.34	0.001
**Prehospital vital signs**					
Hypotension (< 90 mmHg)	0.96	0.92	to	1.01	0.09
**Severity of trauma**					
Revised trauma score	1.20	1.19	to	1.21	< 0.001
AIS					
AIS1 > 3	0.75	0.74	to	0.77	< 0.001
AIS2 > 3	0.76	0.67	to	0.85	<0.001
AIS3 > 3	0.74	0.64	to	0.85	<0.001
AIS4 > 3	0.78	0.76	to	0.79	< 0.001
AIS5 > 3	0.80	0.77	to	0.84	< 0.001
AIS6 > 3	1.40	1.35	to	1.45	< 0.001
AIS7 > 3	1.15	1.10	to	1.21	< 0.001
AIS8 > 3	0.78	0.76	to	0.79	< 0.001
AIS9 > 3	0.74	0.48	to	1.17	0.20
**Interventions before hospital arrival**					
Oxygen	0.89	0.87	to	0.91	< 0.001
Immobilization	1.10	1.08	to	1.12	< 0.001
Chest compression	0.33	0.32	to	0.35	<0.001
Intravenous line placement	0.79	0.73	to	0.85	< 0.001
Defibrillation	0.47	0.37	to	0.60	<0.001
Intubation	0.59	0.52	to	0.66	< 0.001
**Comorbidities**					
Mental disease	1.07	1.02	to	1.11	0.001
Chronic kidney disease on hemodialysis	1.10	1.01	to	1.19	0.04
Malignancy	1.12	1.05	to	1.19	0.001
Diabetes mellitus	1.08	1.05	to	1.12	<0.001
Ischemic heart disease	1.02	0.97	to	1.08	0.38
Chronic heart disease	0.93	0.87	to	1.00	0.06
Cerebrovascular disease	1.01	0.97	to	1.06	0.66
Dementia/mental retardation	0.90	0.86	to	0.94	<0.001

AIS: abbreviated injury scale.

Results of multivariable logistic regression analysis with multiple imputations for missing data comparing factors between patients with shorter on-site time and longer on-site time are reported in Table 3. According to the analysis, the following factors were associated with longer on-site time: younger age (specifically, age 19-59 years was associated with longer on-site time compared to patients aged > 89 years: OR, 1.11; 95% CI, 1.05-1.16; p < 0.001), male sex (OR, 1.14; 95% CI, 1.11-1.16; p < 0.001), more recent trauma (ORs, 1.15-1.42, all p < 0.001), weekends and holidays (OR, 1.06; 95% CI, 1.03-1.08; p < 0.001), alcohol consumption (OR, 1.31; 95% CI, 1.26-1.36; p < 0.001), fall (OR, 1.07; 95% CI, 1.05-1.10; p < 0.001), other blunt trauma (OR, 1.12; 95% CI, 1.07-1.18; p < 0.001), suicide (OR, 1.20; 95% CI, 1.13-1.27; p < 0.001), violence (OR, 1.33; 95% CI, 1.20-1.46; p < 0.001), hypotension (OR, 1.15; 95% CI, 1.09-1.22; p < 0.001), higher RTS (OR, 1.21; 95% CI, 1.20-1.23; p < 0.001), AIS6 ≥ 3 (OR, 1.21; 95% CI, 1.17-1.25; p < 0.001), AIS7 ≥ 3 (OR, 1.15; 95% CI, 1.10-1.21; p < 0.001), immobilization (OR, 1.25; 95% CI, 1.22-1.28; p < 0.001), intravenous line placement (OR, 1.27; 95% CI, 1.17-1.37), intubation (OR, 1.44; 95% CI, 1.26-1.64), mental disease (OR, 1.07; 95% CI, 1.02-1.13; p = 0.003), chronic kidney disease undergoing hemodialysis (OR, 1.12; 95% CI, 1.03-1.22; p = 0.01), and malignancy (OR, 1.11; 95% CI, 1.04-1.18; p = 0.002). Injury during the daytime was associated with shorter on-site time (OR, 0.75; 95% CI, 0.73-0.76; p < 0.001).

**Table 3.** Multivariable Logistic Regression Analysis with Multiple Imputation for Missing Data.

Variables	Odds ratio	95% confidence interval			p-Value
Age groups: years					
19-59			Reference		
60-69	1.00	0.97	To	1.03	0.96
70-79	0.99	0.95	To	1.02	0.37
80-89	0.98	0.94	To	1.01	0.22
89 <	0.90	0.86	To	0.96	< 0.001
Sex					
Male	1.14	1.11	To	1.16	< 0.001
Year					
2004-2007			Reference		
2008-2010	1.15	1.09	to	1.22	< 0.001
2011-2013	1.20	1.14	to	1.26	< 0.001
2014-2016	1.38	1.32	to	1.45	< 0.001
2017-2019	1.42	1.35	to	1.49	< 0.001
Month					
January-March			Reference		
April-June	0.87	0.85	to	0.90	< 0.001
July-September	0.84	0.82	to	0.87	< 0.001
October-December	0.91	0.89	to	0.94	< 0.001
Days of the week					
Weekdays			Reference		
Weekends/Holidays	1.06	1.03	to	1.08	< 0.001
Time					
Daytime	0.75	0.73	to	0.76	< 0.001
Alcohol consumption					
Yes	1.31	1.26	to	1.36	< 0.001
Type of trauma					
Traffic accident			Reference		
Fall	1.07	1.05	to	1.10	< 0.001
Other blunt trauma	1.12	1.07	to	1.18	< 0.001
Penetrating trauma	1.07	0.99	to	1.15	0.09
Cause of trauma					
Accident			Reference		
Occupational injury	0.95	0.91	to	1.00	0.07
Suicide	1.20	1.13	to	1.27	< 0.001
Violence	1.33	1.20	to	1.46	< 0.001
Unclassified/rare mechanisms	1.12	1.01	to	1.25	0.04
**Prehospital vital signs**					
Hypotension (< 90 mmHg)	1.15	1.09	to	1.22	< 0.001
**Severity of trauma**					
Revised trauma score	1.21	1.20	to	1.23	< 0.001
AIS					
AIS1 > 3	0.81	0.79	to	0.84	< 0.001
AIS2 > 3	0.87	0.77	to	0.98	0.02
AIS3 > 3	0.92	0.79	to	1.07	0.28
AIS4 > 3	0.91	0.89	to	0.94	< 0.001
AIS5 > 3	0.86	0.82	to	0.91	< 0.001
AIS6 > 3	1.21	1.17	to	1.25	< 0.001
AIS7 > 3	1.15	1.10	to	1.21	< 0.001
AIS8 > 3	0.81	0.79	to	0.83	< 0.001
AIS9 > 3	0.83	0.52	to	1.32	0.44
**Interventions before hospital arrival**					
Oxygen	0.92	0.90	to	0.95	< 0.001
Immobilization	1.25	1.22	to	1.28	< 0.001
Chest compression	1.07	0.98	to	1.17	0.16
Intravenous line placement	1.27	1.17	to	1.37	< 0.001
Defibrillation	1.03	0.80	to	1.32	0.81
Intubation	1.44	1.26	to	1.64	< 0.001
**Comorbidities**					
Mental disease	1.07	1.02	to	1.13	0.003
Chronic kidney disease on hemodialysis	1.12	1.03	to	1.22	0.01
Malignancy	1.11	1.04	to	1.18	0.002
Diabetes mellitus	1.03	1.00	to	1.07	0.08
Ischemic heart disease	1.04	0.98	to	1.10	0.17
Chronic heart disease	0.98	0.91	to	1.06	0.61
Cerebrovascular disease	1.03	0.98	to	1.08	0.20
Dementia/mental retardation	0.99	0.94	to	1.03	0.56

AIS: abbreviated injury scale.

We performed several sensitivity analyses. First, additional analyses stratified by time period (2004-2007, 2008-2010, 2011-2013, 2014-2016, 2017-2019) showed that the primary associations were largely consistent across all periods (Supplementary Tables 2-6). Male sex, alcohol consumption, RTS, and prehospital interventions such as immobilization and intravenous line placement consistently showed significant associations throughout. In contrast, “Violence” and “Suicide” only became significantly associated in later periods. Second, a sensitivity analysis that excluded patients with cardiac arrest at the scene demonstrated results consistent with the main analysis, with no material changes in the direction or magnitude of the primary associations (Supplementary Table 7). Third, we performed a multivariable logistic regression analysis using a complete-case approach, which excluded patients with missing data. The results of this analysis are presented in Table 4. Fourth, we conducted an additional analysis using the same methodology as the main analysis, but the cutoff for on-site time was redefined as 30 minutes (Supplementary Table 8). These results were largely consistent with those of the primary analysis.

**Table 4.** Multivariable Logistic Regression Analysis with Complete Data.

Variables	Odds ratio	95% confidence interval			p-Value
Age groups: years					
19-59			Reference		
60-69	0.98	0.94	to	1.02	0.34
70-79	0.98	0.94	to	1.03	0.51
80-89	0.96	0.92	to	1.01	0.17
89 <	0.92	0.85	to	1.00	0.047
Sex					
Male	1.08	1.05	to	1.12	< 0.001
Year					
2004-2007			Reference		
2008-2010	1.25	1.17	to	1.36	< 0.001
2011-2013	1.35	1.26	to	1.45	< 0.001
2014-2016	1.41	1.32	to	1.51	< 0.001
2017-2019	1.49	1.39	to	1.60	< 0.001
Month					
January-March			Reference		
April-June	0.85	0.81	to	0.88	< 0.001
July-September	0.82	0.79	to	0.86	< 0.001
October-December	0.90	0.86	to	0.93	< 0.001
Days of the week					
Weekdays			Reference		
Weekends/Holidays	1.07	1.03	to	1.10	< 0.001
Time					
Daytime	0.73	0.71	to	0.75	< 0.001
Alcohol consumption					
Yes	1.30	1.24	to	1.36	< 0.001
Type of trauma					
Traffic accident			Reference		
Fall	1.06	1.02	to	1.10	0.001
Other blunt trauma	0.97	0.91	to	1.04	0.44
Penetrating trauma	0.92	0.82	to	1.02	0.12
Cause of trauma					
Accident			Reference		
Occupational injury	0.99	0.93	to	1.06	0.87
Suicide	1.24	1.14	to	1.36	< 0.001
Violence	1.45	1.27	to	1.66	< 0.001
Unclassified/rare mechanisms	1.43	1.21	to	1.68	< 0.001
**Prehospital vital signs**					
Hypotension (< 90 mmHg)	1.13	1.05	to	1,21	< 0.001
**Severity of trauma**					
Revised trauma score	1.27	1.24	to	1.29	< 0.001
AIS					
AIS1 > 3	0.86	0.83	to	0.89	< 0.001
AIS2 > 3	0.86	0.72	to	1.03	0.10
AIS3 > 3	0.83	0.62	to	1.10	0.19
AIS4 > 3	0.93	0.90	to	0.97	0.001
AIS5 > 3	0.88	0.82	to	0.95	0.001
AIS6 > 3	1.29	1.22	to	1.36	< 0.001
AIS7 > 3	1.23	1.15	to	1.32	< 0.001
AIS8 > 3	0.88	0.85	to	0.92	< 0.001
AIS9 > 3	0.66	0.32	to	1.34	0.25
**Interventions before hospital arrival**					
Oxygen	0.89	0.86	to	0.92	< 0.001
Immobilization	1.20	1.15	to	1.24	< 0.001
Chest compression	1.67	1.24	to	2.24	0.001
Intravenous line placement	1.06	0.91	to	1.22	0.46
Defibrillation	0.56	0.23	to	1.32	0.18
Intubation	1.18	0.84	to	1.66	0.34
**Comorbidities**					
Mental disease	1.07	1.00	to	1.14	0.06
Chronic kidney disease on hemodialysis	1.18	1.04	to	1.33	0.008
Malignancy	1.16	1.06	to	1.26	0.001
Diabetes mellitus	1.00	0.95	to	1.05	0.95
Ischemic heart disease	1.08	1.00	to	1.17	0.04
Chronic heart disease	0.97	0.88	to	1.08	0.62
Cerebrovascular disease	1.12	1.05	to	1.19	0.001
Dementia/mental retardation	1.01	0.94	to	1.08	0.82

AIS: abbreviated injury scale.

## Discussion

To examine factors that contribute to prolonged prehospital on-site time for trauma patients arriving by ambulance, we compared the variables between the shorter on-site time and longer on-site time groups using information from a nationwide trauma database in Japan.

Longer on-site time was associated with the following factors: younger age, male sex, more recent trauma, weekends and holidays, alcohol consumption, fall, other blunt trauma, suicide, violence, hypotension, higher RTS, AIS6 ≥ 3, AIS7 ≥ 3, immobilization, mental disease, chronic kidney disease undergoing hemodialysis, and malignancy.

There are several explanations for these results. Alcohol consumption complicates diagnosis and places a significant burden on healthcare resources and personnel. Both intoxication and withdrawal can obscure or mimic other serious medical conditions, making their assessment and management more difficult ^(23), (24)^. Such patients often require more time and attention during prehospital care, potentially delaying their evaluation and treatment. These challenges continue after hospital arrival, when care for intoxicated patients can be especially demanding, often requiring extended observation, specialized intervention(s), and increased security due to behavioral issues ^(24), (25)^. These factors can hinder hospital acceptance and prolong the time required for EMS to find appropriate receiving facilities. In addition, previous research has shown that stricter enforcement of drinking-and-driving laws is associated with a reduction in traffic-related mortality ^(26)^. These findings suggest that regulatory measures targeting impaired driving may help to reduce both the burden on healthcare systems and negative public health outcomes.

Patients with self-harm, violence-related trauma, and comorbid mental diseases are often difficult to admit due to complex psychiatric conditions, such as personality disorders, post-traumatic stress disorder, or psychosis, which complicate diagnosis and management ^(27), (28)^. Their high risk for recurrence and potential to harm others necessitates intensive monitoring and restrictive measures ^(27), (28)^. In addition, limited resources, staff shortages, and insufficient psychiatric resource capacity make many hospitals hesitant to accept high-risk patients. When psychiatric expertise is required, appropriate intake facilities are often located further away, which prolongs transport time due the need for specialized coordination. A previous study described efforts by tertiary emergency care centers without psychiatric inpatient wards to establish small-scale psychiatric units; these structural changes are expected to have a meaningful impact on the emergency medical system ^(29)^.

Traumas classified as AIS6 (spine) generally do not pose an immediate threat to life but often require transport to specialized facilities to ensure optimal functional prognosis ^(30)^. In Japan, there is a tendency for emergency medical services to select a specialized hospital with surgical and other relevant specialists as the initial destination, especially when advanced treatment is anticipated ^(31)^. This selective process can prolong the time required to decide on a receiving hospital. However, when these injuries coexist with life-threatening trauma, transportation delays must be avoided.

Our findings can be attributed to a combination of clinical procedural needs and systemic vulnerabilities. Clinically, severe upper extremity injuries (AIS 7) often require time-intensive on-site procedures, such as meticulous neurovascular assessment and splinting, which are critical for preserving limb function. This contrasts with life-threatening lower extremity injuries, which are typically managed with a “scoop and run” strategy to prioritize rapid surgical intervention. Systemically, the prolonged on-site time may also reflect the difficulty in securing hospital acceptance, particularly for complex cases like replantable severed fingers, where specialized facilities are required ^(19)^. This interplay between complex on-site care and challenges in hospital selection likely accounts for the observed association. However, these interpretations remain hypothetical, and further studies are needed to confirm these observations.

Our analysis also found that patients with chronic kidney disease undergoing hemodialysis experienced a longer prehospital on-site time. One possible explanation is that patients whose last dialysis session was > 48 hours previously had higher odds for requiring urgent dialysis ^(32)^. In such cases, paramedics may need to coordinate with dialysis-capable facilities to ensure timely treatment, which can lead to additional on-site delays.

There have been no previous studies or plausible explanations regarding ambulance diversion or difficulties in the acceptance of trauma patients with malignant tumors. In this study, an extended on-scene time was observed for trauma patients with malignancy; however, further research is necessary to identify and confirm the underlying causes and reasons for this prolongation.

Higher RTS was associated with longer on-site time. One possible explanation is that the JTDB predominantly includes tertiary care facilities, which may have influenced prehospital transport patterns. Minor injury cases with prolonged on-site time might reflect situations where local hospitals were unable to accept these patients for various reasons, leading to eventual transfer to tertiary centers. However, since the JTDB does not contain information on initial hospital refusals or reasons for interhospital transfer, we could not directly assess this possibility. This limitation should be taken into account when interpreting our findings.

Although multiple factors associated with adverse outcomes have been identified, most are not readily modifiable at the individual patient level, suggesting that patient-specific interventions may have limited effectiveness in improving prognosis. Though some of these factors are clinical in nature, many are inherently patient-specific and difficult to address in prehospital settings. These findings underscore the need for systemic improvements and highlight the potential shortcomings of existing healthcare infrastructure. Future research should focus on evaluating system-level interventions aimed at optimizing care delivery, enhancing interdisciplinary collaboration, and ensuring timely access to appropriate medical resources.

Some differences were observed between the main and sensitivity analyses for certain variables. Nevertheless, these additional analyses further support the robustness of our findings. The temporal stratification analysis confirmed that the main associations were consistent across different time periods (Supplementary Tables 2-6), suggesting that our results are not driven by a specific time frame. The emergence of significant associations for “Violence” and “Suicide” in later years may reflect societal or reporting changes, but does not alter the overall interpretation of the main results. Similarly, the exclusion of patients with cardiac arrest at the scene did not materially affect the results (Supplementary Table 7), reinforcing the stability of the observed associations. The small differences observed between the main analysis and both the complete-case sensitivity analysis (Table 4) and the sensitivity analysis using a 30-minute cutoff for on-site time (Supplementary Table 8) are mainly attributable to the use of multiple imputation in the main analysis versus a complete-case approach in the sensitivity analyses. From a clinical perspective, it is difficult to determine the exact reasons for these discrepancies, which may be influenced by complex interactions among variables and unmeasured confounding factors.

The present study had several strengths. First, to the best of our knowledge, no previous large-scale investigation has examined the factors associated with prehospital on-site time in trauma cases. Second, the nationwide database used in this study included a substantial number of patients, thus enabling the adjustment of numerous factors in the multivariate analysis. Third, we accounted for trauma severity, which is a limitation of previous studies that did not sufficiently incorporate this aspect. Fourth, the dataset does not distinguish whether prehospital interventions were performed at the trauma scene or during transport. Nevertheless, because analyses were adjusted for injury severity and other relevant patient characteristics, and the sample size was large, we believe that the overall patterns remain informative despite this limitation. Fifth, in response to concerns about potential selection bias due to variable inclusion, we ensured that all variables in our model were selected based on their clinical relevance and potential influence on prehospital time, as supported by previous literature. We avoided including variables that may act as mediators in the causal pathway. Additionally, we checked for multicollinearity by assessing correlations and VIFs, which confirmed no problematic relationships among the variables. These steps support the robustness of our findings. Finally, we performed a multivariate analysis with multiple imputations and a complete-case analysis as a sensitivity check, confirming the robustness of our findings.

However, this study also had several limitations. First, detailed reasons for prolonged on-site time remain unclear. Second, despite adjusting for potential covariates, we did not account for several possible residual confounding factors that may affect on-site time, such as regional hospital capacity, occupancy rates, or specific characteristics of the local medical system. For example, regional hospital rotation systems and nighttime emergency coverage schedules may influence hospital availability and prehospital coordination; however, these factors were not captured in the dataset. These structural factors vary across regions and could have affected prehospital time, but were not captured in the dataset. Third, we did not separately analyze on-scene time differences between patients with single injuries and those with multiple injuries. Given that multiple injuries can complicate prehospital management and potentially prolong on-scene time, this distinction is clinically relevant. Future studies should investigate the impact of injury multiplicity on prehospital time to better understand challenges within the Japanese trauma care system. Fourth, given the exploratory nature of this study, further rigorous verification is required for the interpretation of individual factors.

In conclusion, factors associated with prolonged prehospital on-site time for trauma patients included younger age, male sex, more recent trauma, weekends and holidays, alcohol consumption, falls, other blunt trauma, suicide, violence, hypotension, higher RTS, AIS6 ≥ 3, AIS7 ≥ 3, immobilization, mental disease, chronic kidney disease undergoing hemodialysis, and malignancy. Interventions targeting these factors may help shorten prehospital on-site time.

## Article Information

### Acknowledgments

The authors gratefully acknowledge the emergency medical service personnel, nurses, emergency physicians, and healthcare workers who participated in the JTDB (Japan Trauma Data Bank).

### Author Contributions

Conceptualization: Shunichi Otaka, Takuyo Chiba, Takamichi Nakajima, Takashi Shiga. Data curation: Shunichi Otaka. Formal analysis: Shunichi Otaka. Investigation: Shunichi Otaka. Methodology: Shunichi Otaka, Takashi Shiga. Project administration: Shunichi Otaka. Resources: Shunichi Otaka, Takashi Shiga. Software: Shunichi Otaka. Supervision: Takuyo Chiba, Takamichi Nakajima, Takashi Shiga. Writing: Shunichi Otaka. Validation: Shunichi Otaka, Takashi Shiga. Visualization: Shunichi Otaka. Writing - original draft: Shunichi Otaka. Writing - review & editing: Shunichi Otaka, Takuyo Chiba, Takamichi Nakajima, Takashi Shiga. All authors read and approved the final manuscript. Shunichi Otaka had full access to all the data in the study and takes responsibility for its integrity and the data analysis.

### Conflicts of Interest

None

### IRB Approval Code and Name of the Institution

This study was approved by the Institutional Review Board of the International University of Health and Welfare, Narita Hospital (Approval number 22-Im-008; June 28, 2022).

## Supplement

Supplementary Material
