# Supplementary material for: Factors Associated with Prolonged Prehospital On-Site Time in Adult Trauma Patients: A Nationwide Observational Study in Japan
Source: JMA J. 2026 Feb 6;9(2):535–46. doi: 10.31662/jmaj.2025-0290 (PMC13058748; doi:10.31662/jmaj.2025-0290)
Supplement: Supplementary Material [file 2433-3298-9-2_0535-s001.pdf]

Supplementary Table 1. Variance Inflation Factors (VIF) for Independent Variables

Variables	VIF
Age groups: years, n (%)	
19-59	-
60-69	1.23
70-79	1.42
80-89	1.73
89 <	1.42
Sex, n (%)	
Male	1.23
Year, n (%)	
2004-2007	-
2008-2010	2.52
2011-2013	3.61
2014-2016	4.04
2017-2019	3.63
Month, n (%)	
January-March	-
April-June	1.49
July-September	1.50
October-December	1.53
Days of the week, n (%)	
Weekends/Holidays	1.01
Time, n (%)	-
Daytime	1.11
Alcohol consumption, n (%)	-
Yes	1.21
Type of trauma, n (%)	
Traffic accident	-
Fall	1.80
Other blunt trauma	1.40
Penetrating trauma	1.71
Cause of trauma, n (%)	
Accident	-
Occupational injury	1.23
Suicide	1.87
Violence	1.18
Unclassified/rare mechanism	1.02
**Prehospital vital signs**	
Hypotension (< 90 mmHg)	1.78
**Severity of trauma**	

Revised trauma score, median (IQR)	3.21
AIS	
AIS1 > 3, n (%)	1.30
AIS2 > 3, n (%)	1.01
AIS3 > 3, n (%)	1.09
AIS4 > 3, n (%)	1.30
AIS5 > 3, n (%)	1.06
AIS6 > 3, n (%)	1.09
AIS7 > 3, n (%)	1.02
AIS8 > 3, n (%)	1.27
AIS9 > 3, n (%)	1.00
**Interventions before hospital arrival**	
Oxygen	1.62
Immobilization	1.69
Chest compression	2.28
Intravenous line placement	1.16
Defibrillation	1.03
Intubation	1.09
**Comorbidities**	-
Mental disease	1.28
Chronic kidney disease on hemodialysis	1.03
Malignancy	1.02
Diabetes mellitus	1.07
Ischemic heart disease	1.05
Chronic heart disease	1.05
Cerebrovascular disease	1.05
Dementia/mental retardation	1.13
Mean VIF	1.52

AIS: abbreviated injury scale

Supplementary Table 2. (Year 2004-2007) Multivariable Logistic Regression Analysis with Multiple Imputation for Missing Data

Variables	Odds ratio	95% confidence interval			p-Value
Age groups: years					
19-59			Reference		
60-69	0.97	0.86	to	1.10	0.63
70-79	0.88	0.76	to	1.02	0.08
80-89	0.81	0.67	to	0.98	0.03
89 <	0.63	0.44	to	0.89	0.008
Sex					
Male	1.28	1.15	to	1.41	< 0.001
Month					
January-March			Reference		
April-June	0.92	0.81	to	1.04	0.16
July-September	0.78	0.69	to	0.88	< 0.001
October-December	0.85	0.75	to	0.96	0.008
Days of the week					
Weekdays			Reference		
Weekends/Holidays	1.00	0.92	to	1.10	0.95
Time					
Daytime	0.67	0.60	to	0.73	< 0.001
Alcohol consumption					
Yes	1.15	1.01	to	1.32	0.04
Type of trauma					
Traffic accident			Reference		
Fall	1.21	1.07	to	1.37	0.002
Other blunt trauma	1.11	0.92	to	1.35	0.28
Penetrating trauma	0.89	0.68	to	1.17	0.41
Cause of trauma					
Accident			Reference		
Occupational injury	0.97	0.80	to	1.17	0.75
Suicide	1.20	0.97	to	1.48	0.10
Violence	0.91	0.66	to	1.25	0.57
Unclassified/rare mechanism	1.22	0.61	to	2.45	0.57
**Prehospital vital signs**					
Hypotension (< 90 mmHg)	1.14	0.95	to	1.36	0.16
**Severity of trauma**					
Revised trauma score	1.17	1.13	to	1.21	< 0.001
AIS					
AIS1 > 3	0.74	0.66	to	0.82	< 0.001
AIS2 > 3	0.96	0.61	to	1.53	0.88
AIS3 > 3	1.23	0.73	to	2.07	0.44

AIS4 > 3	0.97	0.88	to	1.08	0.60
AIS5 > 3	1.04	0.88	to	1.23	0.63
AIS6 > 3	1.23	1.06	to	1.43	0.006
AIS7 > 3	1.02	0.84	to	1.24	0.84
AIS8 > 3	1.08	0.97	to	1.20	0.15
AIS9 > 3	0.64	0.23	to	1.77	0.39
**Interventions before hospital arrival**					
Oxygen	0.98	0.90	to	0.95	< 0.001
Immobilization	0.99	1.22	to	1.28	< 0.001
Chest compression	1.15	0.98	to	1.17	0.16
Intravenous line placement	1.80	1.17	to	1.37	< 0.001
Defibrillation	1.34	0.80	to	1.32	0.81
Intubation	1.21	1.26	to	1.64	< 0.001
**Comorbidities**					
Mental disease	0.88	0.72	to	1.07	0.19
Chronic kidney disease on hemodialysis	1.10	0.60	to	2.01	0.76
Malignancy	1.38	0.87	to	2.21	0.17
Diabetes mellitus	1.05	0.87	to	1.26	0.60
Ischemic heart disease	1.27	0.94	to	1.72	0.12
Chronic heart disease	0.72	0.43	to	1.21	0.22
Cerebrovascular disease	1.02	0.79	to	1.31	0.89
Dementia/mental retardation	1.07	0.78	to	1.46	0.69

AIS: abbreviated injury scale

Supplementary Table 3. (Year 2008-2010) Multivariable Logistic Regression Analysis with Multiple Imputation for Missing Data

Variables	Odds ratio	95% confidence interval			p-Value
Age groups: years					
19-59			Reference		
60-69	0.96	0.88	to	1.05	0.40
70-79	0.91	0.82	to	1.00	0.05
80-89	0.83	0.74	to	0.94	0.002
89 <	0.63	0.51	to	0.78	< 0.001
Sex					
Male	1.25	1.16	to	1.34	< 0.001
Month					
January-March			Reference		
April-June	0.87	0.79	to	0.96	0.004
July-September	0.88	0.80	to	0.96	0.006
October-December	0.88	0.81	to	0.97	0.007
Days of the week					
Weekdays			Reference		
Weekends/Holidays	1.05	0.98	to	1.12	0.16
Time					
Daytime	0.72	0.67	to	0.77	< 0.001
Alcohol consumption					
Yes	1.31	1.18	to	1.46	< 0.001
Type of trauma					
Traffic accident			Reference		
Fall	1.13	1.04	to	1.23	0.004
Other blunt trauma	1.14	0.99	to	1.32	0.07
Penetrating trauma	1.18	0.96	to	1.45	0.11
Cause of trauma					
Accident			Reference		
Occupational injury	0.99	0.85	to	1.15	0.86
Suicide	1.11	0.95	to	1.30	0.18
Violence	1.11	0.87	To	1.43	0.41
Unclassified/rare mechanism	0.83	0.56	To	1.23	0.36
**Prehospital vital signs**					
Hypotension (< 90 mmHg)	1.16	1.01	to	1.33	0.04
**Severity of trauma**					
Revised trauma score	1.18	1.15	to	1.21	< 0.001
AIS					
AIS1 > 3	0.78	0.72	to	0.84	< 0.001
AIS2 > 3	0.87	0.59	to	1.27	0.46
AIS3 > 3	0.70	0.46	to	1.06	0.09

AIS4 > 3	0.91	0.84	to	0.98	0.02
AIS5 > 3	0.80	0.70	to	0.91	0.001
AIS6 > 3	1.16	1.04	to	1.30	0.008
AIS7 > 3	1.26	1.09	to	1.45	0.002
AIS8 > 3	0.85	0.78	to	0.92	< 0.001
AIS9 > 3	0.86	0.25	to	2.93	0.81
**Interventions before hospital arrival**					
Oxygen	1.06	0.97	to	1.15	0.18
Immobilization	1.14	1.04	to	1.24	0.004
Chest compression	0.98	0.79	to	1.23	0.88
Intravenous line placement	1.56	1.17	to	2.07	0.002
Defibrillation	0.74	0.29	to	1.90	0.54
Intubation	1.23	0.81	to	1.85	0.33
**Comorbidities**					
Mental disease	1.15	1.00	to	1.31	0.050
Chronic kidney disease on hemodialysis	1.04	0.74	to	1.45	0.84
Malignancy	1.23	0.97	to	1.57	0.09
Diabetes mellitus	1.17	1.04	to	1.32	0.008
Ischemic heart disease	0.93	0.77	to	1.12	0.43
Chronic heart disease	0.86	0.65	to	1.13	0.28
Cerebrovascular disease	0.96	0.81	to	1.13	0.60
Dementia/mental retardation	0.97	0.80	to	1.16	0.72

AIS: abbreviated injury scale

Supplementary Table 4. (Year 2011-2013) Multivariable Logistic Regression Analysis with Multiple Imputation for Missing Data

Variables	Odds ratio	95% confidence interval			p-Value
Age groups: years					
19-59			Reference		
60-69	1.01	0.95	to	1.08	0.69
70-79	1.02	0.96	to	1.10	0.48
80-89	0.93	0.87	to	1.01	0.10
89 <	0.91	0.80	to	1.03	0.14
Sex					
Male	1.18	1.13	to	1.24	< 0.001
Month					
January-March			Reference		
April-June	0.90	0.84	to	0.96	0.001
July-September	0.84	0.79	to	0.89	< 0.001
October-December	0.92	0.87	to	0.98	0.01
Days of the week					
Weekdays			Reference		
Weekends/Holidays	1.05	1.00	to	1.10	0.051
Time					
Daytime	0.72	0.69	to	0.76	< 0.001
Alcohol consumption					
Yes	1.35	1.25	to	1.46	< 0.001
Type of trauma					
Traffic accident			Reference		
Fall	1.03	0.97	to	1.09	0.32
Other blunt trauma	1.19	1.07	to	1.32	0.001
Penetrating trauma	1.06	0.91	to	1.23	0.46
Cause of trauma					
Accident			Reference		
Occupational injury	1.01	0.91	to	1.12	0.91
Suicide	1.12	1.00	to	1.26	0.06
Violence	1.59	1.29	to	1.96	< 0.001
Unclassified/rare mechanism	1.34	1.01	to	1.77	0.045
**Prehospital vital signs**					
Hypotension (< 90 mmHg)	1.23	1.11	to	1.37	< 0.001
**Severity of trauma**					
Revised trauma score	1.22	1.20	to	1.25	< 0.001
AIS					
AIS1 > 3	0.83	0.79	to	0.88	< 0.001
AIS2 > 3	0.87	0.68	to	1.11	0.26
AIS3 > 3	0.98	0.73	to	1.32	0.88

AIS4 > 3	0.90	0.85	to	0.95	< 0.001
AIS5 > 3	0.80	0.72	to	0.88	< 0.001
AIS6 > 3	1.28	1.19	to	1.38	< 0.001
AIS7 > 3	1.17	1.05	to	1.29	0.003
AIS8 > 3	0.87	0.82	to	0.92	< 0.001
AIS9 > 3	0.77	0.29	to	2.04	0.60
**Interventions before hospital arrival**					
Oxygen	1.05	0.99	to	1.11	0.09
Immobilization	1.29	1.22	to	1.37	< 0.001
Chest compression	1.06	0.89	to	1.27	0.50
Intravenous line placement	1.58	1.30	to	1.94	< 0.001
Defibrillation	1.17	0.72	to	1.90	0.54
Intubation	1.92	1.45	to	2.56	< 0.001
**Comorbidities**					
Mental disease	1.16	1.06	to	1.29	0.002
Chronic kidney disease on hemodialysis	1.14	0.94	to	1.37	0.18
Malignancy	0.90	0.78	to	1.03	0.13
Diabetes mellitus	0.99	0.92	to	1.07	0.85
Ischemic heart disease	0.95	0.85	to	1.07	0.41
Chronic heart disease	1.00	0.84	to	1.19	0.98
Cerebrovascular disease	1.02	0.93	to	1.13	0.64
Dementia/mental retardation	1.06	0.95	to	1.18	0.32

AIS: abbreviated injury scale

Supplementary Table 5. (Year 2014-2016) Multivariable Logistic Regression Analysis with Multiple Imputation for Missing Data

Variables	Odds ratio	95% confidence interval			p-Value
Age groups: years					
19-59			Reference		
60-69	1.04	0.98	to	1.10	0.20
70-79	1.00	0.94	to	1.06	0.95
80-89	0.99	0.93	to	1.05	0.73
89 <	0.97	0.88	to	1.06	0.46
Sex					
Male	1.09	1.05	to	1.14	< 0.001
Month					
January-March			Reference		
April-June	0.91	0.86	to	0.95	< 0.001
July-September	0.88	0.83	to	0.92	< 0.001
October-December	0.97	0.92	to	1.02	0.19
Days of the week					
Weekdays			Reference		
Weekends/Holidays	1.08	1.04	to	1.12	< 0.001
Time					
Daytime	0.75	0.72	to	0.78	< 0.001
Alcohol consumption					
Yes	1.30	1.21	to	1.40	< 0.001
Type of trauma					
Traffic accident			Reference		
Fall	1.07	1.02	to	1.12	0.009
Other blunt trauma	1.09	1.00	to	1.19	0.046
Penetrating trauma	1.14	1.00	to	1.31	0.053
Cause of trauma					
Accident			Reference		
Occupational injury	0.98	0.89	to	1.07	0.63
Suicide	1.18	1.07	to	1.31	0.001
Violence	1.36	1.14	to	1.61	0.001
Unclassified/rare mechanism	0.98	0.84	to	1.15	0.79
**Prehospital vital signs**					
Hypotension (< 90 mmHg)	1.13	1.02	to	1.25	0.01
**Severity of trauma**					
Revised trauma score	1.22	1.20	to	1.24	< 0.001
AIS					
AIS1 > 3	0.85	0.81	to	0.89	< 0.001
AIS2 > 3	0.83	0.68	to	1.02	0.08
AIS3 > 3	0.92	0.70	to	1.21	0.55

AIS4 > 3	0.94	0.89	to	0.98	0.01
AIS5 > 3	0.88	0.80	to	0.97	0.009
AIS6 > 3	1.20	1.12	to	1.28	< 0.001
AIS7 > 3	1.15	1.06	to	1.25	0.001
AIS8 > 3	0.79	0.75	to	0.83	< 0.001
AIS9 > 3	1.00	0.38	to	2.61	0.99
**Interventions before hospital arrival**					
Oxygen	0.94	0.90	to	0.98	0.01
Immobilization	1.30	1.24	to	1.36	< 0.001
Chest compression	0.86	0.73	to	1.02	0.08
Intravenous line placement	1.34	1.17	to	1.55	< 0.001
Defibrillation	1.42	0.92	to	2.20	0.11
Intubation	1.06	1.18	to	1.86	0.001
**Comorbidities**					
Mental disease	1.06	0.97	to	1.15	0.20
Chronic kidney disease on hemodialysis	1.08	0.93	to	1.26	0.30
Malignancy	1.18	1.06	to	1.32	0.002
Diabetes mellitus	1.00	0.95	to	1.06	0.88
Ischemic heart disease	1.10	1.00	to	1.21	0.045
Chronic heart disease	1.07	0.94	to	1.21	0.30
Cerebrovascular disease	1.10	1.01	to	1.19	0.03
Dementia/mental retardation	1.01	0.93	to	1.10	0.74

AIS: abbreviated injury scale

Supplementary Table 6. (Year 2017-2019) Multivariable Logistic Regression Analysis with Multiple Imputation for Missing Data

Variables	Odds ratio	95% confidence interval			p-Value
Age groups: years					
19-59			Reference		
60-69	0.98	0.92	to	1.04	0.49
70-79	0.99	0.93	to	1.06	0.82
80-89	1.06	0.99	to	1.13	0.09
89 <	0.93	0.84	to	1.02	0.13
Sex					
Male	1.07	1.02	to	1.12	0.003
Month					
January-March			Reference		
April-June	0.81	0.77	to	0.86	< 0.001
July-September	0.82	0.77	to	0.86	< 0.001
October-December	0.87	0.82	to	0.92	< 0.001
Days of the week					
Weekdays			Reference		
Weekends/Holidays	1.05	1.00	to	1.09	0.04
Time					
Daytime	0.78	0.75	to	0.82	< 0.001
Alcohol consumption					
Yes	1.33	1.24	to	1.43	< 0.001
Type of trauma					
Traffic accident			Reference		
Fall	1.07	1.01	to	1.12	0.02
Other blunt trauma	1.08	0.98	to	1.19	0.13
Penetrating trauma	0.99	0.84	to	1.15	0.85
Cause of trauma					
Accident			Reference		
Occupational injury	0.87	0.79	to	0.96	0.01
Suicide	1.34	1.19	to	1.50	< 0.001
Violence	1.42	1.13	to	1.78	0.003
Unclassified/rare mechanism	1.45	1.15	to	1.82	0.001
**Prehospital vital signs**					
Hypotension (< 90 mmHg)	1.08	0.97	to	1.19	0.17
**Severity of trauma**					
Revised trauma score	1.25	1.22	to	1.28	< 0.001
AIS					
AIS1 > 3	0.79	0.75	to	0.83	0.17
AIS2 > 3	0.90	0.71	to	1.14	0.39
AIS3 > 3	0.86	0.63	to	1.18	0.36

AIS4 > 3	0.87	0.82	to	0.92	< 0.001
AIS5 > 3	0.86	0.77	to	0.95	0.005
AIS6 > 3	1.17	1.10	to	1.26	< 0.001
AIS7 > 3	1.10	0.99	to	1.21	0.06
AIS8 > 3	0.73	0.69	to	0.76	< 0.001
AIS9 > 3	0.81	0.27	to	2.43	0.70
**Interventions before hospital arrival**					
Oxygen	0.76	0.73	to	0.80	< 0.001
Immobilization	1.23	1.17	to	1.29	< 0.001
Chest compression	1.28	1.05	to	1.56	0.02
Intravenous line placement	1.19	1.04	to	1.35	0.01
Defibrillation	0.64	0.37	to	1.11	0.11
Intubation	1.43	1.10	to	1.85	0.007
**Comorbidities**					
Mental disease	1.04	0.94	to	1.14	0.45
Chronic kidney disease on hemodialysis	1.18	1.02	to	1.37	0.03
Malignancy	1.15	1.03	to	1.29	0.01
Diabetes mellitus	1.05	0.99	to	1.12	0.13
Ischemic heart disease	1.09	0.98	to	1.20	0.11
Chronic heart disease	0.94	0.82	to	1.06	0.27
Cerebrovascular disease	1.02	0.94	to	1.11	0.64
Dementia/mental retardation	0.90	0.83	to	0.98	0.02

AIS: abbreviated injury scale

Supplementary Table 7. Multivariable Logistic Regression Analysis with Multiple Imputation for Missing Data (Excluding Patients with Cardiac Arrest)

Variables	Odds ratio	95% confidence interval			p-Value
Age groups: years					
19-59			Reference		
60-69	1.00	0.97	to	1.03	0.92
70-79	0.98	0.95	to	1.02	0.29
80-89	0.97	0.94	to	1.01	0.13
89 <	0.90	0.85	to	0.95	< 0.001
Sex					
Male	1.13	1.11	to	1.16	< 0.001
Year					
2004–2007			Reference		
2008–2010	1.17	1.11	to	1.23	< 0.001
2011–2013	1.22	1.16	to	1.28	< 0.001
2014–2016	1.42	1.35	to	1.49	< 0.001
2017–2019	1.46	1.39	to	1.54	< 0.001
Month					
January-March			Reference		
April-June	0.87	0.85	to	0.90	< 0.001
July-September	0.84	0.82	to	0.87	< 0.001
October-December	0.92	0.89	to	0.94	< 0.001
Days of the week					
Weekdays			Reference		
Weekends/Holidays	1.06	1.03	to	1.08	< 0.001
Time					
Daytime	0.74	0.73	to	0.76	< 0.001
Alcohol consumption					
Yes	1.32	1.27	to	1.37	< 0.001
Type of trauma					
Traffic accident			Reference		
Fall	1.07	1.04	to	1.10	< 0.001
Other blunt trauma	1.06	1.00	to	1.11	0.04
Penetrating trauma	1.02	0.94	to	1.10	0.69
Cause of trauma					
Accident			Reference		
Occupational injury	0.97	0.92	to	1.02	0.29
Suicide	1.24	1.17	to	1.32	< 0.001
Violence	1.38	1.25	to	1.53	< 0.001
Unclassified/rare mechanism	1.15	1.03	to	1.28	0.02
**Prehospital vital signs**					
Hypotension (< 90 mmHg)	1.15	1.09	to	1.21	< 0.001

**Severity of trauma**					
Revised trauma score	1.22	1.20	to	1.24	< 0.001
AIS					
AIS1 > 3	0.82	0.80	to	0.84	< 0.001
AIS2 > 3	0.86	0.76	to	0.97	0.02
AIS3 > 3	0.86	0.72	to	1.02	0.08
AIS4 > 3	0.91	0.89	to	0.94	< 0.001
AIS5 > 3	0.85	0.80	to	0.89	< 0.001
AIS6 > 3	1.23	1.19	to	1.28	< 0.001
AIS7 > 3	1.16	1.10	to	1.21	< 0.001
AIS8 > 3	0.81	0.79	to	0.83	< 0.001
AIS9 > 3	0.73	0.44	to	1.19	0.20
**Interventions before hospital arrival**					
Oxygen	0.92	0.90	to	0.95	< 0.001
Immobilization	1.25	1.22	to	1.28	< 0.001
Intravenous line placement	1.19	1.08	to	1.32	0.001
Intubation	1.35	1.12	to	1.63	0.002
**Comorbidities**					
Mental disease	1.07	1.02	to	1.13	0.01
Chronic kidney disease on hemodialysis	1.12	1.03	to	1.23	0.01
Malignancy	1.11	1.04	to	1.18	0.002
Diabetes mellitus	1.03	0.99	to	1.06	0.15
Ischemic heart disease	1.04	0.98	to	1.10	0.19
Chronic heart disease	0.98	0.90	to	1.05	0.53
Cerebrovascular disease	1.04	0.99	to	1.09	0.15
Dementia/mental retardation	0.99	0.94	to	1.04	0.62

AIS: abbreviated injury scale

Supplementary Table 8. Multivariable Logistic Regression Analysis with Multiple Imputation for Missing Data (On-site Time < 30 vs ≥ 30 minutes)

Variables	Odds ratio	95% confidence interval			p-Value
Age groups: years					
19-59			Reference		
60-69	0.94	0.89	to	0,99	0.03
70-79	0.98	0.93	to	1.04	0.56
80-89	0.89	0.84	to	0.95	0.001
89 <	0.79	0.71	to	0.87	< 0.001
Sex					
Male	1.26	1.21	to	1.31	< 0.001
Year					
2004–2007			Reference		
2008–2010	1.36	1.23	to	1.50	< 0.001
2011–2013	1.49	1.36	to	1.63	< 0.001
2014–2016	1.45	1.32	to	1.58	< 0.001
2017–2019	1.22	1.11	to	1.35	< 0.001
Month					
January-March			Reference		
April-June	0.80	0.76	to	0.85	< 0.001
July-September	0.76	0.72	to	0.80	< 0.001
October-December	0.83	0.79	to	0.87	< 0.001
Days of the week					
Weekdays			Reference		
Weekends/Holidays	1.09	1.05	to	1.14	< 0.001
Time					
Daytime	0.67	0.64	to	0.69	< 0.001
Alcohol consumption					
Yes	1.55	1.46	to	1.64	< 0.001
Type of trauma					
Traffic accident			Reference		
Fall	1.13	1.08	to	1.19	< 0.001
Other blunt trauma	1.28	1.18	To	1.39	< 0.001
Penetrating trauma	1.04	0.92	to	1.17	0.57
Cause of trauma					
Accident			Reference		
Occupational injury	1.01	0.93	to	0.93	0.81
Suicide	1.21	1.10	to	1.10	< 0.001
Violence	1.65	1.44	to	1.44	< 0.001
Unclassified/rare mechanism	1.31	1.11	to	1.11	0.001
**Prehospital vital signs**					
Hypotension (< 90 mmHg)	0.99	0.91	to	1.08	0.86

**Severity of trauma**					
Revised trauma score	1.19	1.17	to	1.21	< 0.001
AIS					
AIS1 > 3	0.82	0.78	to	0.86	< 0.001
AIS2 > 3	0.75	0.60	to	0.95	0.02
AIS3 > 3	0.59	0.43	to	0.81	0.001
AIS4 > 3	0.86	0.82	to	0.91	< 0.001
AIS5 > 3	0.78	0.71	to	0.86	< 0.001
AIS6 > 3	1.34	1.26	to	1.42	< 0.001
AIS7 > 3	1.22	1.12	to	1.32	< 0.001
AIS8 > 3	0.91	0.87	to	0.95	< 0.001
AIS9 > 3	1.07	0.49	to	2.34	0.87
**Interventions before hospital arrival**					
Oxygen	0.84	0.81	to	0.88	< 0.001
Immobilization	1.10	1.05	to	1.15	< 0.001
Chest compression	0.95	0.78	to	1.16	0.63
Intravenous line placement	1.87	1.63	to	2.13	< 0.001
Defibrillation	0.86	0.49	to	1.52	0.60
Intubation	1.28	0.97	to	1.67	0.08
**Comorbidities**					
Mental disease	1.14	1.06	to	1.24	0.001
Chronic kidney disease on hemodialysis	1.18	1.01	to	1.36	0.03
Malignancy	0.97	0.87	to	1.09	0.60
Diabetes mellitus	0.96	0.91	to	1.02	0.21
Ischemic heart disease	0.90	0.82	to	1.00	0.046
Chronic heart disease	0.98	0.85	to	1.13	0.80
Cerebrovascular disease	0.94	0.86	to	1.02	0.14
Dementia/mental retardation	1.07	0.98	to	1.17	0.13

AIS: abbreviated injury scale
